# Lie detection algorithms disrupt the social dynamics of accusation behavior

**DOI:** 10.1016/j.isci.2024.110201

**Published:** 2024-06-27

**Authors:** Alicia von Schenk, Victor Klockmann, Jean-François Bonnefon, Iyad Rahwan, Nils Köbis

**Affiliations:** 1 Julius-Maximilians-Universität Würzburg, Department of Economics, Sanderring 2, 97070 Würzburg, Germany; 2 Center for Humans and Machines, Max Planck Institute for Human Development, Lentzeallee 94, 14195 Berlin, Germany; 3 Institute for Advanced Study in Toulouse, Toulouse, France; 4 Toulouse School of Economics, Toulouse, France; 5 Research Center Trustworthy Data Science and Security, University of Duisburg-Essen, Bismarckstrasse 120, 47057 Duisburg, Germany

**Keywords:** Applied computing, Social sciences, Research methodology social sciences

## Abstract

Humans, aware of the social costs associated with false accusations, are generally hesitant to accuse others of lying. Our study shows how lie detection algorithms disrupt this social dynamic. We develop a supervised machine-learning classifier that surpasses human accuracy and conduct a large-scale incentivized experiment manipulating the availability of this lie-detection algorithm. In the absence of algorithmic support, people are reluctant to accuse others of lying, but when the algorithm becomes available, a minority actively seeks its prediction and consistently relies on it for accusations. Although those who request machine predictions are not inherently more prone to accuse, they more willingly follow predictions that suggest accusation than those who receive such predictions without actively seeking them.

## Highlights

• Supervised learning algorithm surpasses human accuracy in text-based lie detection
• Without algorithmic support, people are reluctant to accuse others of lying
• Availability of a lie-detection algorithm increases people’s lying accusations
• 31% of participants request algorithmic advice, among those, most follow its advice

## Graphical abstract

**Figure:**
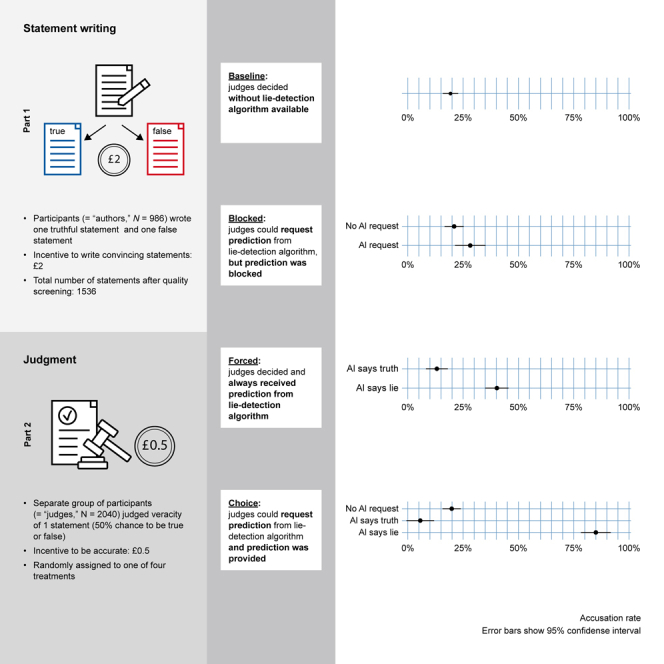

## Introduction

People lie a lot.^1^^,^^2^^,^^3^^,^^4^ In many contexts, such as to detect fake reviews or prevent the spread of “fake news,” it would be advantageous to detect lies and call them out.^5^^,^^6^^,^^7^^,^^8^ Think of a politician making campaign promises in speeches or a salesperson promoting the features of a product in written descriptions. Because the interests of the politician and the salesperson may not be aligned with those of the viewer, it may be challenging for the viewer to decide whether to trust the promises and claims. While some methods help with lie detection,^9^^,^^10^ the time, effort, and skill, they require to place them beyond the reach of ordinary people. Accordingly, recent studies and large-scale meta-analyses indicate that people do not perform much better than chance when trying to detect lies.^11^^,^^12^^,^^13^^,^^14^^,^^15^^,^^16^

This generally poor performance in lie detection may offer insights into why people typically refrain from accusing others of dishonesty.^17^^,^^18^^,^^19^ Truth-default theory (TDT) proposes that when evaluating the truthfulness of others, people typically operate under a default assumption that what they are hearing is true.^20^ According to TDT, this truth-default leads to a general and automatic tendency to believe others. Only under specific conditions, such as when discrepancies or contradictions are noticed, this default to truth is overridden, and skepticism arises. This theory explains why detecting deception is challenging and why people are often susceptible to believing false information, such as the content of deepfake videos.^21^ Indeed, not being able to discern truth from lies increases the risk of making false accusations, which are harmful both to the accused and to the accuser. False accusations can harm the accused because of the social stigma of being called a liar, and they can, in turn, harm the accuser, who is held accountable for unjustly tarnishing the reputation of the accused. Since people are generally bad at detecting lies, it may be a safer strategy to refrain from lying accusations that can hurt both the accuser and the accused if they are unfounded.

As a corollary, anything that would reduce either the harm to the accused or the accountability of the accuser may increase the rate at which people accuse each other of lying. For example, the harm of false accusations to the accused can be reduced by systematic fact-checking. Currently, this time-consuming process is mostly reserved for high-stakes accusations (e.g., in judicial or political contexts) and is unlikely to be available in all accusation contexts (e.g., social media). Technology may change that if fact-checking can be automated and scaled up, but the real technological game changer may consist of automatic lie detection that decreases the accountability of the accuser rather than automated fact-checking that reduces harm to the accused.

Indeed, the progress in artificial intelligence (AI) is opening a new chapter in the long history of lie-detecting machines.^22^^,^^23^ While older machines such as the polygraph have a high degree of noise^24^ and other technologies relying on physical and behavioral characteristics such as eye-tracking and pupil dilation measurements are complex and expensive to implement,^25^ current Natural Language Processing algorithms can detect fake reviews,^5^^,^^26^ spam detection on X/Twitter,^27^ and achieve higher-than-chance accuracy for text-based lie detection.^28^^,^^29^^,^^30^ Indeed, a large collection of algorithms has been developed with the aim of detecting deception and lies^31^^,^^32^^,^^33^ (see for recent reviews).^34^^,^^35^ If this AI technology continues to improve and becomes massively available, it may disrupt the way people largely refrain from accusing each other of lying.

This disruption could have significant policy implications. Imagine a world in which everyone has access to superhuman lie-detection technologies, such as Internet browsers that screen social media posts for lies, algorithms that check CVs for deception, or video conferencing platforms that give real-time warnings when one’s interlocutor or negotiation partner seems to be insincere, as is not unusual in negotiations.^36^ Consulting a lie-detection algorithm, or delegating accusations to the algorithm, could reduce accusers’ sense of accountability, increase the psychological distance from the accused, and blur questions of liability.^37^^,^^38^ As a result, we might witness a rise in accusations, leading to new social, legal, and ethical challenges.

The policy relevance of this research lies in understanding how these potential disruptions may unfold and how individuals will choose to use lie-detection technology. Our results align with past research, showing that people often hesitate to use algorithms, especially when they are not entirely error-proof.^39^ Although there is evidence of a more appreciative view of algorithms in neutral contexts,^40^ the resistance to adopting algorithmic solutions is particularly notable in emotionally charged situations.^41^ This resistance could influence the timing and extent of adopting lie-detection algorithms, consequently affecting the social landscape and prompting considerations for regulations in an increasingly automated world. The economic implications extend to sectors relying on human judgment in lie detection, such as the legal system and human resources.

In this study, we develop a supervised machine-learning lie-detection algorithm. Its accuracy significantly exceeds that of humans. We then conduct an incentivized lie-detection experiment in which we measure participants’ propensity to use the algorithm, as well as the impact of that use on accusation rates and accuracy. To establish the causal effect of the algorithm on behavior, and compare it to endogenous selection into the use of the algorithm, we exogenously manipulate, on the individual level, the choice and availability of the lie-detection algorithm and show that both have an impact on accusation rates.

Our study builds on and extends the nascent research field on hybrid lie detection, particularly the work by Kleinberg and Verschuere.^30^ They were among the first to test how people incorporate lie-detection algorithms in their judgments. The most important differences from their study are that our study incorporates (1) incentives for participants who write the statements to ensure that they are motivated to write convincing texts, (2) a more widely available algorithm, and (3) an experimental design that allows testing who wants to receive algorithmic advice and whether those who do request advice differ in their reliance on the algorithmic predictions from those who do not.

Our results indicate that in a Baseline treatment without the lie-detection algorithm, people are hesitant to make accusations. In a Choice treatment where the lie-detection algorithm is an option, a minority seeks its predictions, and those who do overwhelmingly rely on the algorithm, especially when it recommends accusations. The propensity to request predictions by the lie-detection algorithm does not inherently increase the likelihood of making accusations, as a Blocked treatment shows that those who request but do not receive predictions from the algorithm do not exhibit a significant change in behavior. Similarly, as revealed by a Forced treatment, individuals who do not actively seek predictions from the lie-detection algorithm remain unchanged in their behavior, regardless of receiving passive algorithm-generated predictions. Finally, we find that adoption rates of the lie-detection algorithm are influenced by perceptions of its relative performance.

## Experimental design and data

### Statement writing study

As preparation for our main study, in the Statement Collection Study, we collected a dataset of true and false statements to be used for training the algorithm and for the lie-detection task. We recruited 986 participants via Prolific and asked them to describe something they intended to do during the next weekend. We explicitly chose a neutral and not politically loaded context and a task promising high variation in the answers to allow for extensive training of the algorithm. While some studies let people decide whether to lie or not,^42^^,^^43^ we adopted standard procedures in research on lie detection and elicited true and false statements from each participant.^30^^,^^44^ Participants were first asked to write a true statement together with a supporting text briefly arguing that their statement was indeed truthful. Afterward, they saw the activities of four randomly selected other participants and were asked to indicate which of them they were not going to carry out. One of the selected activities was then picked at random, and participants then wrote a false statement with incentives to lie convincingly (they earned a bonus of £2 if a future participant judged their statement to be true; see details below). They were not informed beforehand that they would have to write a false statement after the truthful one. For the purpose of an earlier version of this paper, half of all authors were given the possibility to block the use of a lie-detection algorithm for their two statements for a fee of £0.30. This has no effect on the statements and is not used further in our analyses. We exclude subjects who picked this option.

This approach has the advantage of obtaining better training data for the lie-detection algorithm because (1) we avoid selection bias of lies stemming from the endogenous choice by participants, and (2) true and false statements are balanced in the training dataset.

Two research assistants coded the quality of these statements. First, after eliminating answers below the requested minimum of 150 characters, they checked whether the participant followed the instructions and wrote meaningful and understandable sentences. Second, for truthful statements, they checked whether the author’s supporting text fitted the statement (see Appendix S2). Participants were excluded from the dataset if at least one of their statements failed at least one of the respective checks. After this quality control, our dataset contained 1,536 statements from 768 authors.

### Lie-detection algorithm

To generate a lie-detection algorithm that performed better than humans, we relied on the open-source BERT language model by Google.^45^ To obtain a prediction of truthfulness for each statement, we repeatedly performed an 80:20 split of the data. Specifically, we first split the 1,536 statements into five equally sized subsets. We then trained the model with 80% of the data and tested it with the remaining 20% and repeated this step five times. Accuracy was then measured by the total performance of the algorithm across the test datasets of all five folds. The algorithm reached 66.86% accuracy (i.e., correctly identifying lies as lies and truthful statements as truth), 63.19% precision (i.e., how many accusations by the algorithm were justified), 80.78% recall (i.e., how many lies were identified as such), and an F1-score of 70.91%. We deemed that sufficient to proceed to the next stage, given the expectation that humans would not perform much better than chance level at this task. More details on the lie-detection algorithm, its implementation, and its performance relative to other classifiers in the literature can be found in Appendices S1.4, S2.1, and S2.2.

### Judgment study

For the main Judgment Study, we selected 510 statements at random while preserving the algorithm’s confusion matrix (see Tables S2 and S3 in the Appendix). For exemplary statements and descriptive statistics see Appendix S1.3. We recruited 2,040 participants (=“judges”) via Prolific ($M_{age}=36.93,SD_{age}=10.63,38.38\%$ female, additional details in Appendix S1.1). In all experimental treatments, each of the participants who acted as a judge read one statement and decided whether this statement was true or false. Judges were incentivized for accuracy (bonus of £0.50 for a correct guess) and were informed beforehand that half of all statements in the underlying dataset were truthful and the other half were lies. They also learned that the statement they would see was picked randomly. To understand the consequences of their decisions, judges learned that the author of the statement would only earn a bonus with certainty when not being accused of lying. This abstracts from the idea that the accusation of dishonesty always harms the accused (e.g., through a loss of reputation), regardless of whether it was justified or not. We explicitly chose a binary choice paradigm for the judges for several reasons. First, implementing a decision upon the probability with which a statement is true or false would introduce a rather complicated incentive framework, which might pose challenges in terms of participant comprehension. Additionally, it would be difficult to implement the payoff consequences of accusations for the accused authors in case of a non-binary judgment. Second, in real-life scenarios, individuals typically do not quantify the probability of someone else’s dishonesty but rather make binary choices to accuse or not.

**Figure 1:**
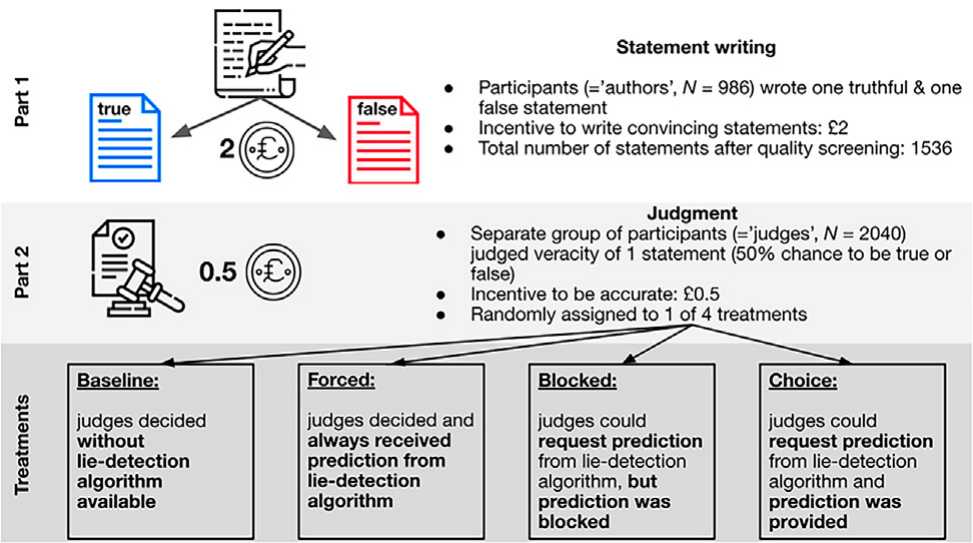
Overview of the study design Notes: the study consisted of two parts. In Part 1, participants (=authors) wrote one true and one false statement. In Part 2, a separate sample of participants (=judges) judged one randomly drawn statement in four different treatments: in the Baseline, judges decided by themselves, without any lie-detection algorithm available; in the Forced treatment, all judges received a prediction from the lie-detection algorithm; in the Blocked treatment, judges could request a prediction from a lie-detection algorithm, but that prediction was blocked; in the Choice treatment judges could request a prediction from a lie-detection algorithm and that prediction was provided.

Judges were randomly assigned on an individual level to one of four treatments that orthogonally manipulated the choice and availability of the lie-detection algorithm. As a result, four between-subjects conditions (baseline plus three treatments) with 510 judges each exist. Namely, in the Baseline treatment, participants judged the statements without any lie-detection algorithm available, and they did not learn about the existence of a lie-detection algorithm until after their judgment. In the Forced treatment, all judges receive the prediction of the lie-detection algorithm. In the Blocked and Choice treatments, the same instructions are identical to the Forced treatment yet judges can request the prediction of the lie-detection algorithm (“true statement” or “false statement”) for a small cost of £0.05. They were informed that there was a chance that the prediction would be blocked (a random draw executed at the beginning of the experiment during treatment assignment). In all cases in the Blocked treatment, the prediction was blocked. Judges were simply informed that the prediction was unavailable (without further elaboration), and their purchase was refunded. Hence, in this treatment, some judges requested but did not receive the prediction from the lie-detection algorithm. In the Choice treatment, all judges who requested a prediction from the lie-detection algorithm received it and were charged the £0.05 cost (see Figure 1 for an overview of the study design). In general, our experimental design is related to Kleinberg and Verschuere.^30^ Yet, we differ mainly in two aspects. First, the predictions of the algorithm are binary. Participants, therefore, need to assess the reliability for themselves. Second, we introduce the endogenous choice to adopt the lie-detection algorithm in the Blocked and Choice treatments.

At the end of the study, all judges answered a series of questions measuring their beliefs about the accuracy of the algorithm (percentage of mistakes, percentage of false accusations, accuracy compared to the average human, accuracy compared to themselves) and feelings of guilt when accusing somebody of lying in this study. We informed those judges who did not have access to the lie-detection algorithm about the existence of an intelligent algorithm that was designed to predict the truthfulness of statements. This elicitation allowed us to assess whether the decision to use the algorithm correlated with subjective expectations about its performance.

### Procedural details

The experiment was programmed in oTree,^46^ and participants were recruited via Prolific. We collected the statement dataset in January 2022 and used the data for training the lie-detection algorithm. We then conducted a first pilot study on the use of lie-detection algorithms in April 2022. The main Judgment Study took place in May 2023, in which judges on average took about 4 min to complete the whole experiment with mean earnings of £1.93. We preregistered our analyses on AsPredicted. All data collections were approved by the Ethics Committee of the Max Planck Institute for Human Development. Participants provided informed consent at the start of the study. Experimental instructions, datasets, STATA analysis scripts for the analyses for the pilot study, the results reported below, as well as the pre-registration (AsPredicted #130240, https://aspredicted.org/wa9mn.pdf) are available on the Open Science Framework (database: https://osf.io/eb59s).

## Results

### Human and algorithmic performance

Judges in the Baseline treatment achieved a 46.47% accuracy rate in judgments, in line with previous findings documenting people’s inability to discern truthful statements from lies.^44^ Further, the accuracy of their accusations (40.82%) was lower but not significantly distinguishable from chance (one-sample t test with $H_{0}:\mu =0.5$, t=−1.84, p=0.07).

Our lie-detection algorithm achieved an overall accuracy rate of 66.86%, which is comparable to previously developed lie-detection algorithms.^22^^,^^30^ It significantly exceeds both random guessing (one-sample t test, t=8.08, p<0.001) and human performance (two-sample t test, t=6.71, p<0.001, Cohen’s d=0.42). As can be seen in the confusion matrix in the Appendix, Tables S2 and S3, we observe higher accuracy for untruthful statements (80.78%) than truthful statements (52.94%).

#### Result 1

People’s accuracy in the Baseline (=without a lie-detection algorithm) does not significantly differ from chance. Our supervised machine-learning lie-detection algorithm significantly exceeds chance levels and outperforms human judgements.

### Lie accusations across treatments

**Figure 2:**
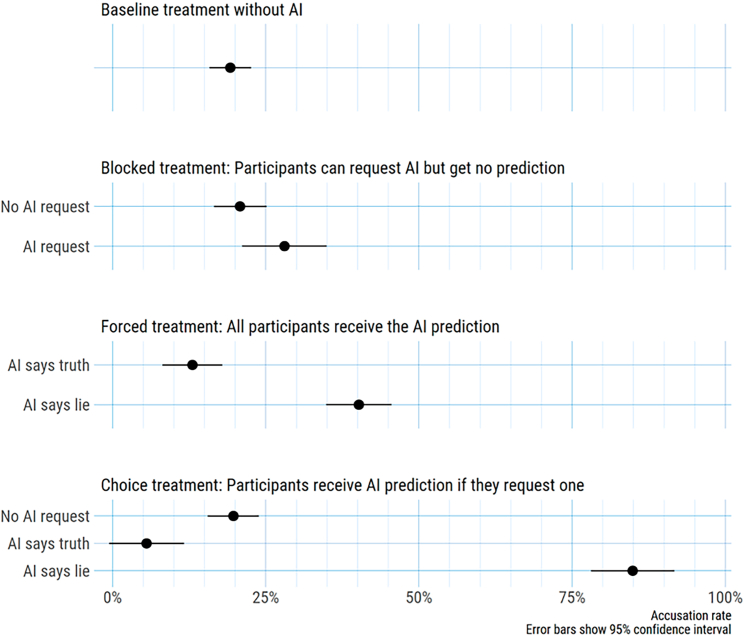
Accusation rates across treatments Notes: the figure plots the estimate (and 95% confidence intervals) of accusation rates across treatments, across request status for algorithmic predictions (request vs. not), and algorithmic prediction type (truth vs. lie).

We analyze accusation behavior separately by treatment as well as, if applicable, by whether the judge requested a hint and by what prediction the lie-detection algorithm made for the given statement. Table 1 reports overall accusations and accusations conditional on the algorithmic prediction for the Forced treatment and for judges in the Choice treatment who purchased a hint. Table 2 summarizes the adoption rate of the lie-detection algorithm in the Blocked and the Choice treatments together with accusation rates contingent on this choice. Figure 2 illustrates the differences in behavior across treatments.

**Table 1:** Accuracy and accusation rates

Treatment	Accuracy	Accusations	Accuracy of accusations	Accusation if Algo predicts lie	Accusation if Algo predicts truth
Baseline	46.47%	19.22%	40.82%	–	–
Forced	56.47%	30.39%	60.65%	40.18%	13.04%
Blocked	48.43%	23.14%	46.61%	–	–
Choice	50.78%	31.76%	51.23%	84.91%	5.56%
Algorithm	66.86%	63.92%	63.19%	–	–

**Table 2:** Accuracy and accusation rates by algorithm usage

Treatment	Request hint	Accusations if hint requested	Accusations if no hint requested	Accuracy if hint requested	Accuracy if hint requested
Blocked	32.16%	28.05%	20.81%	48.78%	48.26%
Choice	31.37%	58.13%	19.71%	58.13%	47.43%

In the Baseline treatment (= no lie-detection algorithm available), the accusation rate was 19.22%, even though judges knew that 50% of all statements in the underlying database were lies. This finding confirms the assumption that people typically refrain from accusing others of lying.^17^

In the Blocked treatment, the overall accusation rate was 23.14%, and the overall accuracy (48.43%) was again not different from chance level (one-sample t test, t=−0.71, p=0.48). We see that 32.16% of judges requested a prediction (but did not obtain it). Accusation rates were not significantly distinguishable for those who did (28.05%) and those who did not request a prediction from the lie-detection algorithm (20.81%) ($\chi^{2}=3.28$, p=0.07). Accuracy rates and accuracy of accusations between those who requested (and were denied) algorithmic predictions and those who did not request do not differ significantly (accuracy 48.78% vs. 48.27%, $\chi^{2}=0.01$, p=0.91; accuracy of accusations 41.30% vs. 50.00%, $\chi^{2}=0.85$, p=0.36). There is thus no strong evidence for a systematic difference between algorithm adopters and non-adopters regarding their accusation behavior. Further, there is no significant difference in accusations between the Blocked treatment and the Baseline treatment ($\chi^{2}=2.35$, p=0.13). The mere information on the existence of an algorithm thus does not affect choices.

#### Result 2

People who actively request predictions from the lie-detection algorithm are not inherently more prone to make lying accusations compared to those who do not request algorithmic predictions.

In the Forced treatment, where all judges passively received algorithmic predictions, the overall accusation rate was 30.39%, which significantly exceeds the accusation rate in the Baseline treatment ($\chi^{2}=17.08$, p<0.001, Cramér’s V=0.13). Overall accuracy in this treatment (56.47%) significantly exceeds chance level (one-sample t test, t=2.94, p=0.003), and the accuracy of accusations increased even further to 60.65%, also significantly higher than chance level (one-sample t test, t=2.70, p=0.008). We observed a strong asymmetry in the degree judges adopted the prediction of the lie-detection algorithm, depending on when the algorithm prediction says “lie” or “truth.” When the machine-learning classifier predicted that the statement is true, 86.96% of judges adopted this prediction, yet when the algorithm predicted a lie, only 40.18% of judges adopted this prediction ($\chi^{2}=105.02$, p<0.001, Cramér’s V=0.45).

As a consequence, accusation rates significantly differ too: when the algorithm predicted “truth,” the accusation rate was merely 13.04%, whereas when it predicted “lie,” it rose to 40.18% ($\chi^{2}=40.95$, p<0.001, Cramér’s V=0.28). This finding suggests that when, by default, people receive recommendations from a lie-detection algorithm, they tend to follow them more when such compliance does not require accusation.

#### Result 3

Observing the prediction of the lie-detection algorithm significantly increases accuracy and accusations. People follow algorithmic recommendations significantly more often when it predicts truth rather than lie.

Our last treatment provides an opportunity to see whether this asymmetry holds for participants who actively request a prediction from the lie-detection algorithm instead of passively receiving it. In the Choice treatment, where participants received a prediction from the lie-detection algorithm only if they actively requested and purchased it, around 31.37% of participants made that choice, which replicates the 32.16% uptake observed in the Blocked treatment ($\chi^{2}=0.07$, p=0.79). The overall accusation rate was 31.76%. This accusation rate was significantly higher than in the Baseline treatment ($\chi^{2}=21.14$, p<0.001, Cramér’s V=0.14) but not higher than in the Forced treatment ($\chi^{2}=0.22$, p=0.64). The overall accuracy in this treatment (50.78%) and the accuracy of accusations (51.23%) was not different from chance level (one-sample t tests, overall accuracy: t=0.35, p=0.72; accuracy of accusations: t=0.31, p=0.75).

Requesting and receiving the algorithm’s prediction affected accusation rates. Namely, the accusation rate among those who did not request machine predictions was 19.71%. This accusation rate does not differ from the accusation rates in the Baseline treatment ($\chi^{2}=0.03$, p=0.86) or among those who did not request machine predictions in the Blocked treatment ($\chi^{2}=0.13$, p=0.72). However, when participants requested and received a prediction, their accusation rate significantly increased to 58.13% ($\chi^{2}=74.74$, p<0.001, Cramér’s V=0.38). In contrast to the Forced treatment, participants in the Choice treatment did not display a significant asymmetry in compliance with the different types of predictions of the lie-detection algorithm. Namely, when the algorithm predicted the statement was true, 94.44% of participants adopted that prediction, and 84.91% of participants adopted the prediction of the algorithm when it predicted a lie ($\chi^{2}=3.11$, p=0.08).

As a consequence, accusation rates are strongly shaped by the content of the prediction (5.56% when the prediction is “truth” vs. 84.91% when the prediction is “lie”; $\chi^{2}=92.55$, p<0.001, Cramér’s V=0.76). It shows that people are even more willing to follow algorithmic predictions when they had previously requested them (88.13%), compared to when they just received them as in the Forced treatment (57.06%) ($\chi^{2}=51.32$, p<0.001, Cramér’s V=0.28). This difference stems largely from those who receive “lie” predictions who are significantly more likely to follow the prediction in the Choice treatment than in the Forced treatment (84.91% vs. 40.18%, $\chi^{2}=64.03$, p<0.001, Cramér’s V=0.39).

#### Result 4

About one-third of the people request the prediction of the lie-detection algorithm. The vast majority of those who decided to purchase the recommendation followed the algorithmic advice, independent of whether it predicted truth or lie.

These results thus provide additional perspective on the results of the Forced treatment. In the Forced treatment, we observed an overall accusation rate of 30.39%. Suppose that about 30% of participants would have requested a prediction from the lie-detection algorithm if they had had a choice (cf. results in the Blocked and Choice treatment) and that these participants had made about 60% accusations overall (cf. results in the Choice treatment for algorithm adopters). In this case, one obtains the results of the Forced treatment only if those 70% of participants who received a prediction, but would not have asked for it, accused with 20% probability as in the baseline. That is, these 70% disregarded the algorithmic prediction completely. These findings support that actively choosing and receiving algorithmic predictions results in substantially higher accusation rates.

### Predictors of algorithmic uptake

Across both treatments, we observe algorithmic uptake levels of around 32%. Arguably, such low uptake levels undermine the effect that algorithms have on social interactions. We analyze several predictors of uptake to find out which ones correlate with the decision of using the lie-detection algorithm and to anticipate the magnitude of the social changes they might provoke.

At the end of the experiment, we asked judges five exploratory questions to gain a better understanding of who decides to request a prediction from the lie-detection algorithm. Two questions asked for a subjective estimation of the absolute performance of the algorithm, namely, its accuracy (0%–100%) and the probability that its accusations are wrong (0%–100%). Two questions asked for a subjective estimation of the comparative performance of the algorithm: whether it would be better than the average human (on a scale from −5, Human’s performance is better, to +5, Algorithm’s performance is better) and whether it would be better than the participant himself or herself (on a scale from −5, My performance is better, to +5, Algorithm’s performance is better). Finally, we asked how much guilt the participant would feel about making a wrong accusation (on a scale from −5, not guilty, to +5, very guilty).

Table 3 displays the average marginal effects of logistic regression models predicting the frequency at which judges elect to use the algorithm as a function of their four judgments about its expected performance and feelings of guilt (restricted to the Blocked and Choice treatments). Because the responses to the four performance questions were all correlated (0.20–0.65 range), we do not include them simultaneously in a single regression model. First, we see no significant link between uptake and neither the belief about the algorithm’s general predictive accuracy nor guilt. Second, the other three measures significantly correlate with algorithmic use in the intuitive direction. Namely, people are more willing to request algorithmic predictions when they believe (1) it outperforms an average human, (2) it outperforms themselves, and (3) the probability of false accusations is low. The missing correlation between beliefs in the algorithm’s overall accuracy and the willingness to use it can be explained by a highly significant, positive correlation between Belief Accuracy and Belief Own vs. Algo (Spearman-ρ of 0.2121,p<0.001). This means that those who believe that the algorithm performs well are at the same time convinced of their own ability to outperform the algorithm and, therefore, prefer to make their own judgments.

**Table 3:** Predictors of algorithm usage

	(1)	(2)	(3)	(4)	(5)
Belief Accuracy	−0.0474 (0.0745)				
Belief False Accusation		−0.1569∗ (0.0703)			
Belief Average vs. Algo			0.2803∗∗∗ (0.0624)		
Belief Own vs. Algo				0.1945∗∗ (0.0598)	
Guilt					−0.0118 (0.0442)
Number of Observations	1,020	1,020	1,020	1,020	1,020
Log-Likelihood	−637.38	−635.11	−627.82	−632.39	−637.55

Notes: the table reports average marginal effects from logistic regressions of the frequency of requesting a hint in the Blocked and Choice treatments on elicited beliefs and feelings of guilt, normalized on the unit interval: the belief about the general accuracy of the algorithm, its false accusation rate, its performance compared to an average human (from 0, the human is better, to 1, the algorithm is better), its performance compared to the participant (from 0, oneself the algorithm is better, to 1, the algorithm oneself is better), and feelings of guilt when accusing somebody of lying in the study (from 0, not guilty, to 1, very guilty). Standard errors are reported in parentheses. Significance coding: ∗ p<0.05, ∗∗ p<0.01, ∗∗∗ p<0.001.

To assess their economic significance, we calculate the changes in the likelihood of purchasing the hint when the respective belief increases by one standard deviation. For the belief in the algorithm’s performance relative to the average human (sd=0.230), the probability increases by 6.45 pp; for the belief in the algorithm’s performance relative to oneself (sd=0.242), the probability increases by 4.71 pp; and for the belief on the algorithm’s false accusation rate (sd=20.70), the probability of using the algorithm decreases by 3.25 pp. Taken together, we find that beliefs about the relative performance of the lie-detection algorithm and of its error rate, not the beliefs about the algorithm’s performance in general or subjective feelings of guilt, predicted adoption rates.

## Discussion

Today, we live in a world where false accusations of lying come with costs, both to the accuser and the accused. One important feature of this world is that people generally refrain from accusing others of lying.^20^ Here, we were interested in understanding the potential impact of novel AI-driven lie-detection technologies on this social behavior.

Specifically, we hypothesized that technologies that lower the probability or the cost of false accusations might increase the rate at which people accuse each other of lying. We introduce a version of such future technologies into an incentivized experiment, where a lie-detection algorithm significantly outperforms humans in detecting lies in written statements. To understand how such technology affects the social dynamics of lie detection and accusations, we manipulated the choice and availability of predictions by a lie-detection algorithm.

The aggregate results across treatments suggest the following conclusions: (1) in the absence of a lie-detection algorithm, people are reluctant to make accusations; (2) when the lie-detection algorithm is available, a minority of people want to obtain its prediction; (3) the minority that does almost always follows the algorithmic prediction, independent of whether the algorithm flags a statement as truth or lie; (4) individuals who actively request algorithmic predictions are not inherently more prone to make accusations, but follow accusation suggestions more than those who receive such predictions without actively seeking them; (5) those who would not actively request the algorithmic prediction do not change their behavior even when they passively receive one; (6) beliefs about the relative performance of the lie-detection algorithm correlate with adoption rates.

We find that people are overall reluctant to accuse others of lying, especially when no lie-detection algorithms are available. This finding supports the truth-default theory and replicates commonly observed findings in the lie-detection literature, documenting that people typically refrain from accusing others of lying.^17^^,^^18^^,^^20^ One potential reason is that they are simply not very good at it and want to reduce the risks of paying the costs of false accusations for themselves and the accused. In support of this notion, in our study, people also did not succeed at reliably discerning true from false statements.

The supervised machine-learning algorithm we used, however, did manage to exceed chance levels at this task, hence providing a less labor-intensive alternative to heuristic-based approaches that have achieved higher-than-chance levels at lie detection.^10^ Intuitively, it would thus make sense for people to use it. However, only approximately one-third of the participants decided to do so. This reluctance to use algorithmic predictions, even when they can improve human decisions, aligns with a rich literature on algorithm aversion that has revealed such reluctance across various domains.^47^ We add to this literature by documenting algorithm aversion in the context of lie detection, thereby also informing discussions around data protection policies, which are integral to economic activities in the digital age.

Moreover, we find an indication that people who are less averse to algorithmic predictions are not more likely to accuse others of lying. Namely, accusation rates in the Blocked treatment did not differ between those who requested but did not receive predictions by the machine-learning classifier and those who did not request predictions. We thus find no evidence that potential algorithm adopters are inherently more likely to make accusations. It appears instead that for those who are willing to use lie-detection algorithms, the availability of predictions raises their willingness to accuse others of lying.

If a lie-detection algorithm provides a prediction per default, people incorporate this prediction into their judgment, replicating previous work showing that people follow algorithm-generated advice in morally sensitive decisions.^48^ However, people’s reliance on suggestions by a supervised machine-learning algorithm is weaker than when they actively request the prediction. Passive recipients of algorithmic predictions who would not have requested any recommendation remain unchanged in their behavior. Interestingly, the Forced provision of the algorithmic predictions is the only treatment in which the overall accuracy levels exceed chance levels, again underlining that algorithm aversion in the Choice treatment mitigates the impact of lie-detection algorithms. While mandatory machine predictions increase overall accuracy, requested predictions have a stronger influence on people’s willingness to follow these predictions. The vast minority actively asking for a recommendation of the lie-detection algorithm also consistently relies on it for accusations.

This downstream effect of requested algorithmic predictions becomes particularly apparent for the accusation rate. When people sought algorithmic predictions, and the lie-detection algorithm flagged a statement as a lie, accusation rates climbed to almost 85%. One plausible explanation is that an available lie-detection algorithm offers the opportunity to transfer the accountability for accusations from oneself to the machine-learning system.^37^^,^^49^ However, when participants can use an algorithm for lie detection, they rely more on its recommendations when they believe it makes more accurate predictions compared to their own assessment. This finding suggests that in a morally controversial domain such as lie detection, algorithmic uptake is not purely driven by blame-shifting motives but also by the desire to rely on algorithmic support to make more accurate and fair judgments. Delegating a decision involving as much as calling someone a liar to an algorithm without a secure fact-checking process at least invokes considerations about the predictive power and reliability of such systems.

Our results suggest that lie-detection algorithms could have a strong disruptive potential. One possibility is that high accusation rates may strain our social fabric by fostering generalized distrust and further increasing polarization between groups that already find it difficult to trust one another. However, making accusations easier, especially if these accusations are reasonably accurate, may also lead to beneficial effects by discouraging insincerity and promoting truthfulness in personal and organizational communications. Accuracy is an important factor here: individuals can easily get false confidence in their ability to detect lies. Such is the case when they are exposed to pseudo-scientific methods of spotting liars (e.g., after learning the techniques of the TV show “Lie to me”^50^). An advantage of lie-detection algorithms is that they can be properly tested and certified for above-human accuracy in a specific domain.^51^

### Limitations of the study

One limitation of our study is that we study a technology that is currently not (widely) available in this form. We acknowledge that lie-detection algorithms, if and when they become available to consumers, will likely come in a range of prices. Our results are restricted to a single and modest price point. Still, a good direction for follow-up research would be to vary the cost of using the algorithm from zero to significantly higher costs than our current £0.05 and track the effect of this cost on adoption and compliance. It is possible, for example, that a zero-cost algorithm may attract more users but also decrease compliance since the user simply tries the algorithm for free without a psychological commitment to use its predictions.

More generally, estimating the positive and negative social effects of lie-detection algorithms is not easy in a controlled online experiment since these effects may unfold slowly, in a cumulative manner, over a long period. Incentivized behavioral experiments are not the best tool for estimating these long-term cumulative effects, which is one limitation of our current work. But even if we cannot fully assess the magnitude and probability of these social changes, it seems reasonable to accept that to maintain a positive balance between benefits and costs, we will need to be mindful of the performance of lie-detection algorithms before making them massively available, and to use them responsibly as individuals and organizations, taking into account their limitations.

### Practical implications

Our findings provide at least an encouraging signal in that direction: algorithmic uptake depends on the perceived accuracy of algorithms compared to humans and oneself and on beliefs about the likelihood of false accusations. This finding suggests that individuals may be mindful of the performance of lie-detection algorithms and use them somewhat responsibly to make accusations.

Organizations, on the other hand, may not always be so careful. Some managerial domains, such as negotiations with suppliers or clients, might be early adopters of lie-detection algorithms and pressure other domains, such as human resources, to do the same. In healthcare, for example, algorithms could be integrated to support patient assessment or the review of insurance claims. In education, algorithms are already used to identify cases of academic dishonesty.^52^ Since suspicion about out-groups may be more socially tolerated than suspicion within the in-group, using lie-detection algorithms when dealing with other organizations, or external entities may pave the way for their use within an organization—the pilot tests using algorithms with asylum seekers by the European Border Agency Frontex might be a harbinger for similar attempts to come.^53^ As AI technologies become more integrated into everyday life, understanding their broader societal implications, including shifts in trust and the attribution of blame, becomes paramount.^54^ Behavioral science has a crucial role in anticipating these dynamics and carefully managing the transition to a society characterized by increased accusations.

### Conclusion

In conclusion, our research underscores the urgent need for a comprehensive policy framework to address the impact of AI-powered lie-detection algorithms. These algorithms significantly increase accusation rates by those who are open to these technologies, even when many are still reluctant to use them. Policymakers should consider measures to protect consumer privacy and trust, reconsider the legal framework for accusations, and promote responsible use of AI, especially in areas such as healthcare and education. Economic policy could consider the incentives or disincentives for adopting AI-powered lie-detection technology, especially in sensitive contexts. This relates to issues of legal liability, public trust, and the consequences of (false) accusations. In addition, this study highlights the importance of addressing the ethical considerations around using AI in lie detection. A proactive approach to shaping the policy landscape in this area will be critical to harnessing the potential benefits of these technologies while mitigating their risks.

## STAR★Methods

### Key resources table

**Table:** 

REAGENT or RESOURCE	SOURCE	IDENTIFIER
**Deposited data**		

Data of experiment	This paper	https://osf.io/53eb8
STATA code for analyses	This paper	https://osf.io/9zcxd

**Software and algorithms**		

oTree	Chen et al.^46^	https://doi.org/10.1016/j.jbef.2015.12.001
BERT	Devlin et al.^45^	https://doi.org/10.48550/arXiv.1810.04805

**Other**		

Preregistration	This paper	https://aspredicted.org/wa9mn.pdf
Experimental instructions	This paper	https://osf.io/mn7kg

### Resource availability

#### Lead contact

Further information and requests for resources and reagents should be directed to and will be fulfilled by the lead contact, Nils Köbis, (nils.koebis@uni-due.de; n.c.kobis@gmail.com).

#### Materials availability

All instruction texts of the materials used are available on the Open Science Framework (https://osf.io/mn7kg).

#### Data and code availability

• Data of the main experiment: https://osf.io/53eb8.
• TATA code to reproduce the statistical analyses and figures: https://osf.io/9zcxd.
• Any additional information required to reanalyze the data reported in this paper is available from the lead contact upon request.

### Experimental model and study participant details

For the experiment reported in this manuscript, we recruited human participants from the online platform Prolific.co that is specialized in enabling high-quality online behavioral research. As specified in our pre-registration (see https://aspredicted.org/wa9mn.pdf), we recruited 2040 participants with US nationality in total for all treatment conditions. The average age of the final sample was $M_{age}=36.93$ ($SD_{age}=10.63$ of which 38.38% self-identified as female. The average duration of the study was about 4 minutes, and each participant received £1.20 as a participation fee, plus potential bonuses based on their behavior in the tasks. The Ethics Committee of the Max Planck Institute for Human Development approved the study design. We obtained informed consent from all participants prior to participation.

### Method details

We used oTree^46^ to program the study. The experiment consisted of the following steps.

1. Participants read an information sheet outlining that:
  • The study would last between 5 and 8 minutes and entails a base pay of £1.20 with the possibility to earn an additional bonus,
  • Participants need to be above 18 years old and their anonymity was ensured,
  • The study was funded by the Max Planck Institute for Human Development,
  • Participants could end the study at any point but would not receive payment in this case, and
  • To participate in the study, they needed to actively consent.
2. Participants read the instructions to the study, informing them that:
  • They would read a short statement by a past participant of this study about a non-work-related activity that is either truthful or a lie,
  • Their task was to guess whether it is truthful or a lie which would earn them a bonus of £0.50 if correct,
  • The authors would lose part of his/her pay if a lie accusation was made,
  • Half of all statements were truthful and half of them were lies, though the statement we showed them would be randomly selected, and
  • In all treatments except the baseline (see below), there is a the lie detection algorithm with higher average performance than humans, and there is the fee of £0.05 to purchase a hint (in treatments Blocked and Choice, see below).
3. We randomly assigned participants to one of four treatments, namely:
  (a) The Baseline in which participants decided without a lie-detection algorithm available,
  (b) The Forced treatment in which participants always received a prediction from the lie-detection algorithm,
  (c) The Blocked treatment in which participants could request a prediction, but it was blocked, and
  (d) The Choice treatment in which participants could request a prediction and received it.
4. After engaging in one practice trial with blind text, participants saw a randomly selected statement and
  • Guessed the truthfulness of the statement and
  • Had the possibility to request a hint (in treatments Blocked and Choice).
5. After the judgment, participants were informed about:
  • Whether they guessed correctly or not and
  • The resulting payment, taking into account the base pay, the potential bonus, and the potential fee for a hint.
6. Participants answered five questions about their perception of the algorithm about:
  • Its overall accuracy in %,
  • The average human performance compared to the algorithm on a Likert scale from -5 to 5,
  • The own performance compared to the algorithm on a Likert scale from -5 to 5,
  • The frequency in % with which the algorithm incorrectly predicts a lie although it is actually a true statement, and
  • How guilty they would feel if authors lost some of their payment because they clicked on “Lie” although the statements were true, on a Likert scale from -5 to 5.
7. Participants answered several exit questions, assessing their:
  • Age (in years),
  • Gender (male, female, or other/non-binary),
  • Highest obtained level of education (No degree, High school, Bachelor, Master, PhD),
  • Major if they went to university (Not applicable, Economics, Law, Psychology, Political sciences, Medicine, Natural sciences, Engineering, Other social sciences, Other),
  • Employment status (Unemployed, Part-time, Full-time), and
  • Familiarity with new technologies such as machine learning (Not familiar at all, Rather not familiar, Neutral, A little familiar, Very familiar).

### Quantification and statistical analysis

We conducted all statistical analyses in STATA. All statistical details and sample sizes are provided. The exact statistical tests and variables used are described in the text and the legends of the tables and figures.

### Additional resources

The study was pre-registered at AsPredicted (see https://aspredicted.org/wa9mn.pdf).

## Supplemental information

Document S1. Tables S1–S5 and Appendices S1 and S2
