# Supplementary material for: Lie detection algorithms disrupt the social dynamics of accusation behavior
Source: iScience. 2024 Jun 27;27(7):110201. doi: 10.1016/j.isci.2024.110201 (PMC11302104; doi:10.1016/j.isci.2024.110201)
Supplement: Document S1. Tables S1–S5 and Appendices S1 and S2 [file mmc1.pdf]

iScience, Volume *27*

Supplemental information

Lie detection algorithms disrupt the social dynamics of accusation behavior

Alicia von Schenk, Victor Klockmann, Jean-François Bonnefon, Iyad Rahwan, and Nils Köbis

Lie Detection Algorithms Disrupt the Social Dynamics of Accusation Behavior

Supplemental Information

S1 Additional Details on the Experimental Design

S1.1 Balance

Table S1 shows summary statistics of several demographic variables across treatments. Chi-squared tests reveal no significant differences in the share of females, the share of participants without university education, the share of fully employed subjects, and the degree of familiarity with technologies such as AI ($p > 0.28$ in any case). There are only significant, though small, differences in age for the Choice treatment.

Table S1: Balance between Treatments. Overview of the demographics across treatments, related to Figure 2

Treatment	Age	Female	No university	Full employment	Familiarity with AI
Baseline	36.15	0.3686	0.3902	0.6725	2.70
Forced	36.75	0.4118	0.3647	0.6157	2.62
Blocked	36.84	0.3784	0.3686	0.6471	2.65
Choice	37.97	0.3765	0.3941	0.6353	2.73

Notes: Averages of demographics across treatments. Familiarity with AI ranges from 0 to 4.

S1.2 Inclusion Criteria for Statements

The research assistants checked whether the authors wrote a meaningful statement about their activities (or, for the false statements, as if they were going to carry it out) as intended. For the truthful statements, they further verified that the additional question asking for supportive information fitted and reinforced the participant's entry. The third criterion was automatically applied and flagged all statements with less than 150 characters. If at least one statement of the authors failed at least one verification, we took out this author completely and did not use any of his/her statements for Part 2.

S1.3 Details on Statements and Examples

Participants were first asked to write a true statement together with a supporting text briefly arguing that their statement was indeed truthful. Afterward, they were shown three activities of other participants of the study and should indicate which of these activities do not apply to them and that they will not carry out. Since those activities were often very specific, for instance, “Cutting some tile to finish the shared bathroom”, “Friend’s mom’s surprise birthday party”, “Making my own game”, we perceive it as very unlikely that plans change. The 510 randomly selected statements for the main Judgment Study contained on average about 370.4 characters and 73.2 words. The shortest statement in the corpus contained 28 words, and the longest was 372 words. There are no significant differences in the number of characters and the number of words between truthful and false statements (373.7 vs. 367.1 characters, $t = 0.55$, $p = 0.58$; 73.5 vs. 72.9 words, $t = 0.26$, $p = 0.80$).

Table S2: Exemplary Statements. Overview of the activities, actual statements and their truthfulness of statements generated in the statement writing task, related to Figure 1

Activity	Description	Truthful
Project of building a new PC	This weekend I am building out my new PC with components I ordered and have been receiving in the mail all week. I just started a new side hustle doing renderings for architectural firms and I need a more powerful PC to handle the heavy graphics processing I will be doing. I may or may not also be using this rig for a little VR video game fun as well haha.	No
Project of building a new PC	All the parts I’ve ordered should be arriving within this week and should take about a day or two days depending if everything goes well. The parts I order includes the Power Supply Unit, CPU, GPU, SDD, HDD, M.2 SSD, motherboard, 32 GB ddr4 RAM, PC case, Computer Monitor, Logitech Mouse, Corsair K95 Keyboard . For software I bought Windows 10 Pro.	Yes
Trip to Disney World	I’m trying to get ready for a trip to Disney World. It’s a bit of a pain. I have to go through all these mental checklists of things that I may need to bring with me, as I am a meticulous planner. I also have to check the weather, as here in North Carolina we have been getting ice storms that shut down the airport, which could throw my whole trip into chaos. I’m trying to make sure we have proper transportation once we arrive as well.	No
Trip to Disney World	I am going to the Festival of the Arts at Epcot in Walt Disney World on Sunday. I am going to try a lot of the different foods in all the different countries. I also want to ride my favorite, Soarin, where you get to virtually travel to different countries. I’m very excited to visit the aquarium they have in the park as well.	Yes
Visiting my grandparents in Florida	I will be visiting my grandparents in Florida next week for about five days. My grandma’s birthday is next week and she will be turning 89. I haven’t seen them in a while so i thought this was a good opportunity to go visit them and see my other cousins living there as well. We don’t really have anything planned but we’re planning to have a nice dinner somewhere.	No
Visiting my grandparents in Florida	I live in the Northeast and will soon be visiting my grandparents in Florida. My grandmother’s birthday is a few days from now, so we want to be there to celebrate this occasion with her. They moved to Florida about 7 years ago and I am excited to see them soon. I haven’t gotten to see them much during the pandemic.	Yes

Table S2: Exemplary Statements (cont'd)

Activity	Description	Truthful
Eating out with my sister in law	i have not seen my sister in law for a while now. we need some time to catch up, so we decided to meet for dinner. we have not decided on a restaurant to go to yet. we will most likely go to olive garden or outback steakhouse. we both like these places and enjoy going there. we will go and have a few drinks, eat dinner, and maybe get some dessert.	No
Eating out with my sister in law	My sister in law is going to be coming to visit me next Wednesday after work. We are going to go to Taco Bell and pick up lunch and bring it back to my house. After lunch we are going to work on crafts which is diamond painting. We enjoy talking and working on our diamond paintings together. It is a craft activity that we share together. She will stay for a few hours and then go home after.	Yes
Flu Shot for Infant	Last week, I was sick with the flu. Luckily, my wife and infant daughter did not get sick. However, this was a wake up call for the both of us. This weekend, we plan for our daughter to get a flu shot (the two of us will get ours shortly after, likely at CVS). We've already schedules an appointment to do so, and now all we need to do is show up on Saturday.	No
Flu Shot for Infant	On Monday, January 31st at 10:00am EST, I will take my 7.5 month old son to his pediatrician to receive his 2nd dose of his flu shot. He has received vaccines at his 2 month, 4 month and 6 month appointments. His 6 month checkup also included his first dose of the flu shot and we were told that he would have to come back a month later to receive his 2nd dose.	Yes
Friday Night Paint Club	Every month before Covid, a group of friends I went to school with would get together at someone's house every week for a girls' night. Usually this would be painting and drink wine because we are artists/ designers and would bounce ideas off of each other. Obviously after Covid happened we couldn't do this any more. After we couldn't stand missing out on this, we decided to make it a Zoom meeting once a week to make up for cabin fever and lost time.	No
Friday Night Paint Club	I love oil painting, and spend Friday nights indulging in art with my friends. We gather for a light meal, set up our canvases and talk about the 'good old times' in art school. I started working on a large format landscape scene. We listen to instrumental jazz and I drift away, deep in thought. I feel the tension melt away with every brushstroke. This is my private time to relax and get away from the hectic life of the city. I use vibrant, complementary colors to inspire a feeling of action and movement in the work. That canvas is the first of a new series of paintings for an upcoming show: Confrontational Landscapes.	Yes

S1.4 Framing and Implementation of the Lie Detection Algorithm

The algorithm is described to participants in all treatments except in the Baseline (where no algorithm is available) as a "state-of-the-art artificially intelligent lie detection algorithm" (see experimental instructions). This notion can be subject to individual interpretation of participants. Still, this description should not bias our experimental results. First, "state-of-the-art" most likely increases participants' general assessment of the algorithm's performance. Nevertheless, in the Choice and Blocked treatments, we observe only little uptake, which would supposedly be even lower with a more neutral framing. Second, our wording should not affect judges' perception of potential performance differences of the algorithm between true and false statements. However, we observe in the Forced treatment a strong asymmetry in the likelihood of following the algorithm's prediction between "AI says truth" and "AI says lie" (see Figure 2 and Result 3 in the main text).

We programmed the algorithm in Python using the transformers package and the pre-trained model BERT[1]. After tokenization of the text corpus (i.e., the written true and false statements of the authors in the Statement Writing Study), we relied on a BERT Model transformer with an added sequence classification head layer (a linear layer on top of the pooled output). We use the BertTokenizer and TFBertForSequenceClassification from the transformers package. For more information, please refer to the package documentation by Hugging Face (https://huggingface.co/docs/transformers/v4.38.2/en/model_doc/bert). To get predictions for each statement, we trained in total five models via cross-validation, where each fold retained 20% of the data as the test sample. For optimization, we relied on the Adam algorithm by Kingma and Ba[2] to minimize the cross-entropy employed as the loss function. Due to the double randomization in the Statement Writing Study (random draw of other participants from whose activities the subject was supposed to indicate the ones s/he would not carry out, and random draw of one of the selected others' activities for writing the made-up statement), the activities were rather balanced between true and false ($\chi^2 = 390.33$, $p = 0.79$). In fact, only six activities are part of the corpus more than twice: Hiking, Hockey Game, Managing Church Finances, Running, Skiing, and Therapy. Therefore, the algorithm is very unlikely to learn to predict the truthfulness of a statement by its frequency in the text corpus.

S2 Additional Details on Lie Detection Performance

S2.1 Human and Algorithmic Performance Measures

Table S3: Human and Algorithmic Performance Overview. Human and Algorithmic Performance in terms of accuracy, precision, recall and F1-score across treatments, related to Table 1 and Table 2

Treatment / Model	Accuracy	Precision	Recall	F1-score
Baseline	46.47%	40.82%	15.69%	22.66%
Forced	56.47%	60.65%	36.86%	45.85%
Blocked	48.43%	46.61%	21.57%	29.49%
Choice	50.78%	51.23%	32.55%	39.81%
Algorithm	66.86%	63.19%	80.78%	70.91%

Notes: The measures for precision and recall consider lies as the positive class.

S2.2 Algorithmic Performance

We illustrate the performance of the lie-detection algorithm used in this task for truthful and untruthful statements with a confusion matrix with the absolute numbers (Table S4) as well as relative frequencies (Table S5).

Table S4: Confusion Matrix in Absolute Numbers. Table showing the absolute numbers of true and false positive as well as the true and false negative classifications by the algorithm, related to the STAR methods

	Statement is untruthful	Statement is truthful
Prediction = untruthful	206	120
Prediction = truthful	49	135

Table S5: Confusion Matrix in Relative Frequencies. Table showing the relative proportions of true and false positive as well as the true and false negative classifications by the algorithm, related to the STAR methods

	Statement is untruthful	Statement is truthful
Prediction = untruthful	40.39%	23.53%
Prediction = truthful	9.61%	26.47%

The algorithm reached 66.86% accuracy. Understanding lies as the positive class, it reached 63.19% precision, 80.78% recall, and an F1-score of 70.91%.

Accuracy and precision are comparable to the nine BERT models developed in Fornaciari et al.[3] who report values between 64.91% and 71.61% and between 61.38% and 66.84%, respectively. The recall of the algorithm implemented for this study, however, lies above the values in

Fornaciari et al.[3] that range between 51.78% and 67.46%. One reason for the different performance can be the different composition of the training sets. While the statements we collected are by design equally split in true and false, non-false texts are the majority class with 68.66% in the dataset used by Fornaciari et al.[3].

Pasquali et al.[4] employed a humanoid robot called iCub in a game in which human participants needed to describe cards to the robot who judged the veracity of the human statements. Using real-time data of players' pupil dilation, the system detected lies with an accuracy of 70.8%, a precision of 73.6%, a recall of 57%, and an F1-score of 64.2%. Compared to the purely text-based algorithm used in our study, iCub shows similar accuracy and higher precision, though lower recall.

References

[1] Jacob Devlin, Ming-Wei Chang, Kenton Lee, and Kristina Toutanova. BERT: Pre-training of deep bidirectional transformers for language understanding. arxiv preprint, 2018. http://arxiv.org/abs/1810.04805.

[2] Diederik P Kingma and Jimmy Ba. Adam: A method for stochastic optimization. arxiv preprint arxiv:1412.6980, 2014. http://arxiv.org/abs/1412.6980.

[3] Tommaso Fornaciari, Federico Bianchi, Massimo Poesio, Dirk Hovy, et al. Bertective: Language models and contextual information for deception detection. In *Proceedings of the 16th Conference of the European Chapter of the Association for Computational Linguistics: Main Volume*. Association for Computational Linguistics, 2021.

[4] Dario Pasquali, Davide Gaggero, Gualtiero Volpe, Francesco Rea, and Alessandra Sciutti. Human vs robot lie detector: Better working as a team? In *International Conference on Social Robotics*, pages 154–165. Springer, 2021.
